# Prognostic relevance of radiological findings on early postoperative MRI for 187 consecutive glioblastoma patients receiving standard therapy

**DOI:** 10.1038/s41598-024-61925-3

**Published:** 2024-05-14

**Authors:** Alexander Malcolm Rykkje, Jonathan Frederik Carlsen, Vibeke Andrée Larsen, Jane Skjøth-Rasmussen, Ib Jarle Christensen, Michael Bachmann Nielsen, Hans Skovgaard Poulsen, Thomas Haargaard Urup, Adam Espe Hansen

**Affiliations:** 1 Department of Radiology, Rigshospitalet, Copenhagen, Denmark; 2 Department of Neurosurgery, Rigshospitalet, Copenhagen, Denmark; 3 Department of Oncology, Rigshospitalet, Copenhagen, Denmark; 4 The DCCC Brain Tumor Center, Rigshospitalet, Copenhagen, Denmark; 5 Department of Clinical Medicine, University of Copenhagen, Copenhagen, Denmark

**Keywords:** CNS cancer, Brain imaging, Magnetic resonance imaging

## Abstract

Several prognostic factors are known to influence survival for patients treated with IDH-wildtype glioblastoma, but unknown factors may remain. We aimed to investigate the prognostic implications of early postoperative MRI findings. A total of 187 glioblastoma patients treated with standard therapy were consecutively included. Patients either underwent a biopsy or surgery followed by an early postoperative MRI. Progression-free survival (PFS) and overall survival (OS) were analysed for known prognostic factors and MRI-derived candidate factors: resection status as defined by the response assessment in neuro-oncology (RANO)-working group (no contrast-enhancing residual tumour, non-measurable contrast-enhancing residual tumour, or measurable contrast-enhancing residual tumour) with biopsy as reference, contrast enhancement patterns (no enhancement, thin linear, thick linear, diffuse, nodular), and the presence of distant tumours. In the multivariate analysis, patients with no contrast-enhancing residual tumour or non-measurable contrast-enhancing residual tumour on the early postoperative MRI displayed a significantly improved progression-free survival compared with patients receiving only a biopsy. Only patients with non-measurable contrast-enhancing residual tumour showed improved overall survival in the multivariate analysis. Contrast enhancement patterns were not associated with survival. The presence of distant tumours was significantly associated with both poor progression-free survival and overall survival and should be considered incorporated into prognostic models.

## Introduction

Glioblastoma is the most aggressive primary brain cancer in adults. The standard therapy, comprising surgery plus radiotherapy with adjuvant and concomitant temozolomide, has improved the prognosis. Since 2005 and despite numerous clinical trials the prognosis remains poor with a median overall survival of 16–20 months^1–4^. However, the prognosis for the individual patients varies significantly. Among other known factors O^6^-methylguanine-DNA methyltransferase MGMT status, age, performance status, steroid use, multifocal disease, and resection versus no resection have been shown to influence survival and may be used to personalise patient treatment^5–10^. Nevertheless, decisive yet unknown prognostic factors may remain. The extent of surgical resection and its effect on survival have been extensively discussed in the literature. Gross total resection has been shown to improve patient prognosis in meta-analyses, while other studies have shown inconsistent impacts on survival, but the quality of the supporting evidence suffers from a lack of randomised studies^5,11–14^.

Magnetic resonance (MR)-imaging is the diagnostic backbone for the evaluation of glioblastoma treatment response including the assessment of surgical outcomes^2^. On early postoperative MR imaging, the radiologist usually categorises contrast-enhancing residual tumour surrounding the resection cavity as either measurable or non-measurable based on measurements of two perpendicular diameters larger or smaller than 10 mm on axial slices^15^. This method of categorising residual tumour is recommended by the response assessment in neuro-oncology (RANO)-working group to serve as a baseline for future evaluations of treatment response. However, to our knowledge, this widely accepted classification scheme has not been given much consideration in the context of survival analysis^16^. Moreover, contrast enhancements bordering the resection cavity on the early postoperative MRI can have a variety of appearances, including but not limited to linear, nodular, or more diffuse, which may in some cases be surgically induced^17–22^. Contrast-enhancing lesions may also be present distant from the area of resection. Those features of postoperative contrast enhancements are not accounted for in the RANO-working group classification scheme but may be an important prognostic factor, although evidence is limited^23^.

The aim of this study was to study the prognostic impact of postoperative MR imaging in a consecutive cohort of patients treated with standard therapy. Specifically, we will assess the RANO-working group classification scheme as well as characterise different types of contrast enhancements bordering or distant from the resection cavity on early postoperative MRI after glioblastoma surgery.

## Methods

### Patient population

All patients over the age of 18 with primary histology-verified glioblastoma, IDH-wildtype (meeting the 2021 WHO classification of CNS tumours^24^) and treated with standard therapy between November 2016 and March 2020 at Rigshospitalet, Copenhagen, Denmark were included in this study. Patients either received surgery followed by an early postoperative MRI or a diagnostic stereotactic biopsy if surgery was not indicated. All patients receiving postoperative MRI were also included in a previous study by Rykkje et al.^21^.

### Ethical approval

The study adhered to the Declaration of Helsinki and Danish regulation, receiving approval from the Capital Region’s Committee on Health Research Ethics (H-19054690). Patient consent was waived by the committee, given the retrospective collection of data on patients with a poor prognosis.

### Postoperative MRI

Postoperative MRI was acquired using 1.5 and 3 Tesla scanners. Scans were obtained between 1 and 3 days following surgery and predominantly on day 2 (median = 45.9 h after surgery). Before contrast injection, the sequences were 2D sagittal T1-weighted (spin echo, 5 mm slice thickness) and 2D coronal T2-weighted FLAIR (4- or 5-mm slice thickness). The sequences after contrast injection with Gadovist (Bayer AB) were 2D axial T2 (radial sampling with Blade/Propeller, 5 mm slice thickness) and 3D sagittal T1 (gradient echo, 1 mm slice thickness). All post-contrast T1 images were evaluated in the axial plane following reconstruction from the 3D acquisition.

### Image candidate factors

The candidate factors derived from the early postoperative MRI for survival analyses were (1) resection status according to the RANO classification scheme, (2) different patterns of contrast enhancement surrounding the surgical cavity and (3) the presence of a distant tumour component. Preoperative MRI was available for all patients as a reference. The data acquired on the resection status and the different contrast enhancement patterns are the same as in a previously published paper by Rykkje et al. and we refer to this paper for a detailed description of the methodology for these factors^21^. All MRIs were analysed by a radiologist with three years of experience (A.M.R.), who was blinded to the time from surgery to the early postoperative MRI and was guided by two neuroradiologists with 12 (J.F.C.) and 25 (V.A.L) years of experience.

#### Resection status

Resection status on early postoperative MRI was defined as either no contrast-enhancing residual tumour, non-measurable contrast-enhancing residual tumour, or measurable contrast-enhancing residual tumour in accordance with the RANO classification. A measurable tumour is defined using T1-weighted images as a contrast-enhancing residual tumour larger than 10 mm on two perpendicular diameters, which must not include cystic cavities and needs to be visible on two or more axial slices^15^. For non-measurable contrast-enhancing residual tumours, at least one of the diameters is less than 10 mm. The remaining patients were categorised as having no contrast-enhancing residual tumour.

#### Contrast enhancement patterns

The morphology of T1-weighted contrast enhancements surrounding the resection cavity was registered for all patients without measurable or distant tumours. The patterns were no enhancement, thin linear (< 3 mm), diffuse, thick linear (> 3 mm), and nodular (< 10 mm). These categories were inspired by previous studies^18,22,25^ and used in our previous publication^21^. If more than one type of enhancement was present in one patient, the results were stratified in order from what was perceived as less tumour-specific (no enhancement) to the more tumour-specific (nodular), so that each patient was represented once.

#### Distant tumour

A distant tumour was defined as a contrast-enhancing tumour at least 1 cm apart from the border of the resection cavity regardless of resection status. This definition of distant tumours did not take into account whether two foci were interconnected by a T2/FLAIR signal.

Multifocal disease on the preoperative MRI, defined as two or more contrast-enhancing tumours at least 1 cm apart and not connected by a T2-FLAIR signal has been identified as a prognostic factor^5^. Therefore, both distant tumours on postoperative MRI and multifocal disease on preoperative MRI were evaluated as model covariates.

### Treatment and follow-up

All patients received standard oncological therapy following the Stupp protocol^3^. Treatment consisted of radiation therapy (total 60 Gy, 5 fractions per week for 6 weeks) and concomitant chemotherapy (75 mg temozolomide per m^2^ of body surface per day, 7 days per week for the duration of radiotherapy). After radiotherapy followed 6 cycles of adjuvant temozolomide (first cycle with 150 mg/m^2^ and the remaining cycles with 200 mg/m^2^).

All patients were followed and evaluated by MRI every 3 months until death or termination of follow-up as described previously^5^. At the time of recurrence, patients who maintained a performance status ≤ 2 were considered for salvage tumour resection and/or second-line treatment.

### Model covariates

Data for modelling included known prognostic factors MGMT status, age, corticosteroid use and ECOG performance status, and multifocal disease. MGMT promoter methylation status was determined by using pyrosequencing on purified DNA with the Therascreen MGMT Pyro Kit (Qiagen). This analysis was performed on all diagnostic samples, including stereotactic biopsies, and mean methylation above 10% was considered positive. More details of the biomarker analysis are described in a previous paper by Abedi et al.^5^. The Eastern Cooperative Oncology Group (ECOG) performance status scale was used to assess the functional status of each patient, ranging from 0 for fully active to 5 for diseased, and was registered at the start of concomitant treatment^26^. Patients receiving at least 10 mg/day of prednisolone when beginning concomitant treatment were corticosteroid users.

### Statistical analyses

Overall survival (OS) was defined as the time from the start of standard oncological therapy with radiation therapy and concomitant chemotherapy to death, and progression-free survival (PFS) was defined as the time from the start of standard oncological therapy to MR-verified progression or death. Data were censored if patients were still alive or lost to follow-up, while missing values would exclude patients from that analysis.

The Kaplan–Meier method with a log-rank test was employed to plot survival curves.

The Cox proportional hazards model was used for modelling survival endpoints, and the results are presented as hazard ratios (HR) with 95% confidence intervals (CI). Assessment of the model assumptions was performed using martingale residuals. Candidate factors (resection status with 4 categories including biopsies as reference, contrast enhancement patterns and distant tumour) were screened for association with PFS and OS and factors with p < 0.10 were considered for multivariate analysis. All known prognostic factors (MGMT status, age, corticosteroid use, performance status, and multifocal disease) were considered for the analysis. The relationship between multifocal disease and distant tumours was assessed using rank correlation analysis. The concordance index (C-index) was calculated as a measure of discrimination^27^. Tenfold cross-validation was applied to the analysis of overall as well as progression-free survival to assess the estimated model with correction for optimism. The significance level was set to 5%. All statistical calculations were made using SPSS, SAS (v9.4, SAS Institute, Cary, N.C., USA) and R (v 3.1.0 R Development Core team, Vienna, Austria, http://www.R-project.org) (package RMS).

## Results

### Patient cohort

A total of 187 glioblastoma patients treated with standard therapy were included (Table 1). The median age was 59 years (range: 18–75), and the gender distribution was 56% male and 44% female. Tumour resection was carried out in 160 patients (86%) and was followed by postoperative MRI in 153 patients (96% of resected), while 7 had no postoperative MRI. The remaining 27 patients (14%) underwent a diagnostic biopsy instead of surgery. At the time of initiation of concomitant radio-chemotherapy, most patients (94%) had a good performance status (0–1), and almost half of the patients (51%) were still treated with corticosteroids. The MGMT promoter was methylated in 103 patients (45%).

**Table 1:** Patient characteristics (*n* = 187).

Age (years), median (range)	59 (18–75)	Corticosteroid use, n (%)	
Gender, n (%)		Yes	95 (51)
Female	82 (44)	No	91 (49)
Male	105 (56)	Missing	1
Tumour resection, n (%)		ECOG performance status, n (%)	
Resection (with postoperative MRI)	153 (82)	0	110 (59)
Resection (no postoperative MRI)	7 (4)	1	66 (35)
Biopsy	27 (14)	2	7 (4)
Resection status^a^, n (% of 153 with postoperative MRI)		Missing	4 (2)
No enhancing residual tumour	50 (33)	MGMT status, n (%)	
Non-measurable residual tumour	84 (55)	Methylated	85 (45)
Measurable residual tumour	19 (12)	Unmethylated	102 (55)
Distant tumour, n (% of 153)		Multifocal disease, n (%)	
Yes	22 (14)	Yes	27 (14)
No	131 (86)	No	160 (86)

*MRI* magnetic resonance imaging, *MGMT* O^6^-methylguanine-DNA methyltransferase, *ECOG* eastern cooperative oncology group.

^a^Assessed by MRI, usually within 72 h after surgery.

**Figure 1:**
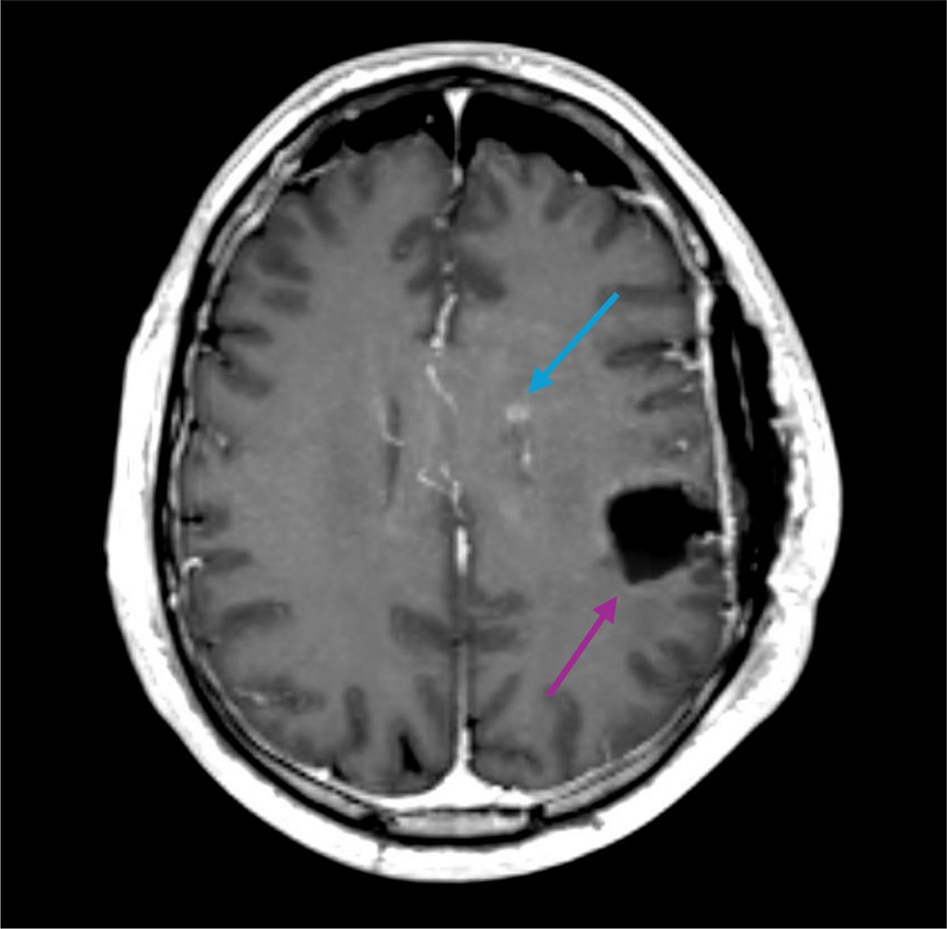
Axial gadolinium-enhanced T1-weighted image of a patient with a distant tumour (yellow arrow). The distant tumour is seen anteromesial to the resection cavity (blue arrow).

On preoperative MRI, 27 of 187 patients (14%) had multifocal disease. Of the 153 surgically resected patients with postoperative MRI, 19 patients (12%) had measurable contrast-enhancing residual tumours according to the RANO classification, 84 (55%) had non-measurable contrast-enhancing residual tumours, and 50 (33%) had no contrast-enhancing residual tumours. A distant tumour was present in 22 (14%) of the 153 patients with an early postoperative MRI (example shown in Fig. 1). Contrast enhancement patterns were analysed for the 118 patients without any measurable or distant tumour on postoperative MRI and were stratified in order of perceived severity: 21 had no contrast enhancements, 30 had only thin linear enhancements, 9 had diffuse (and thin) enhancements, 24 had thick linear (and diffuse and/or thin) enhancements, and 34 had nodular (and diffuse and/or thick or thin linear) enhancements.

At first recurrence, 63 patients (34%) underwent renewed surgical resection and 2^nd^ line treatment was administered in 129 patients (69%). At the time of analysis, 20 (11%) patients were alive out of which 10 patients were without progression. 167 (89%) patients had deceased. Data on time to progression was missing for three patients and five patients were excluded from the multivariate analysis due to missing values as detailed in Table 1. The median follow-up time from the date of treatment initiation was 53 months (range 38–68 months).

### Univariate analyses of progression-free and overall survival

The median progression-free survival was 7.2 months (95% CI 6.5–7.9), while the overall median survival was 17.3 months (95% CI 14.8–19.7). Progression-free survival and overall survival for all factors shown in Table 1 and contrast enhancement patterns were assessed with univariate analyses and are presented in the supplementary document.

Candidate factors associated with poor progression-free survival by univariate analysis were the presence of distant tumours (HR = 2.49, 95% CI 1.51–4.11, p < 0.001), while improved progression-free survival was observed for no contrast-enhancing residual tumour after surgery compared to biopsy only (HR = 0.64, 95% CI 0.40–1.04, p = 0.07) and for non-measurable contrast-enhancing residual tumour compared to biopsy only (HR = 0.63, 95% CI 0.40–0.98, p < 0.04). No significant associations with progression-free survival were observed for the different types of contrast enhancement patterns when compared with MRI with no enhancement (thin linear, diffuse, thick linear, nodular; p > 0.10) (Supplementary Table S1).

Candidate factors associated with poor overall survival were distant tumours (HR = 2.85, 95% CI 1.77–4.59, p < 0.001), while improved overall survival was near-significant for patients with non-measurable contrast-enhancing residual tumour (HR = 0.68 [0.43–1.09], p = 0.11). No significant association with overall survival was observed for the different types of contrast enhancement patterns (p > 0.10) (Supplementary Table S1).

Among the known prognostic factors, methylated MGMT exhibited a significant association with both improved progression-free survival (HR = 0.50, 95% CI 0.37–0.69, p < 0.001) and improved overall survival (HR = 0.46, 95% CI 0.33–0.63, p < 0.001) in the univariate analysis. Increased age was only associated with improved overall survival (HR = 0.80, 95% CI 0.68–0.94, p = 0.01). Multifocal disease was associated with poor progression-free survival (HR = 1.77, 95% CI 1.15–2.72, p = 0.01) and overall survival (HR = 2.28, 95% CI 1.49–3.47, p < 0.001) (Supplementary Table S2).

**Figure 2:**
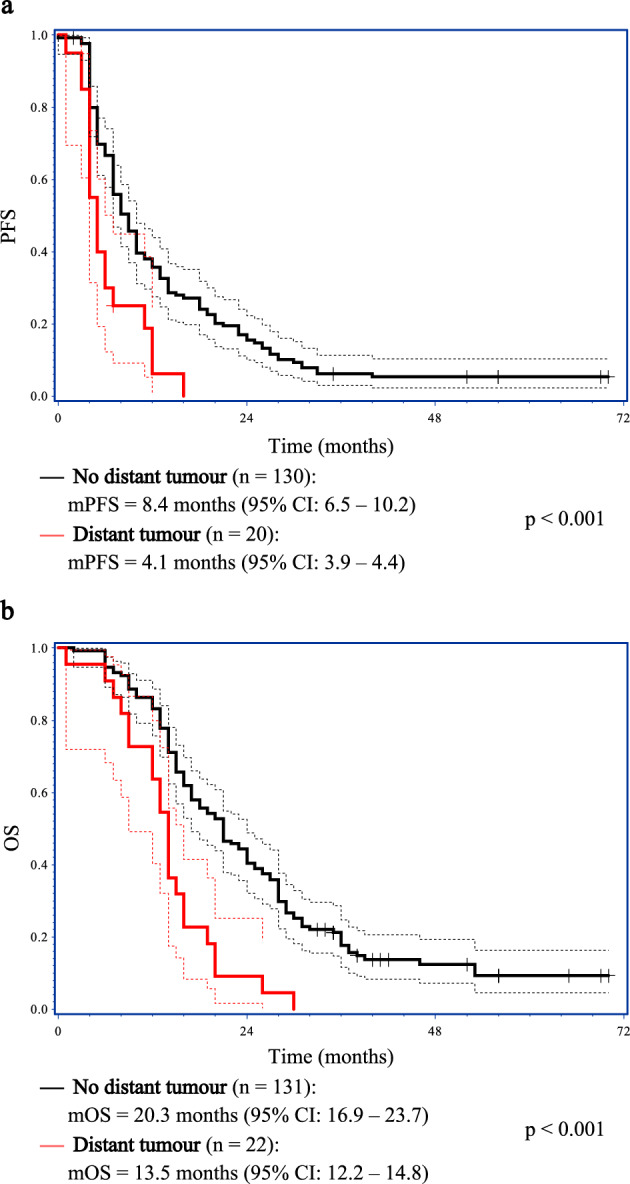
Kaplan–Meier plots with median progression-free survival (mPFS) (**a**) and median overall survival (mOS) (**b**) for patients with or without distant tumour. Dotted lines represent the 95% confidence intervals (CI), while vertical markers indicate censored patients. Median PFS and OS with 95% CIs are presented below each plot.

**Figure 3:**
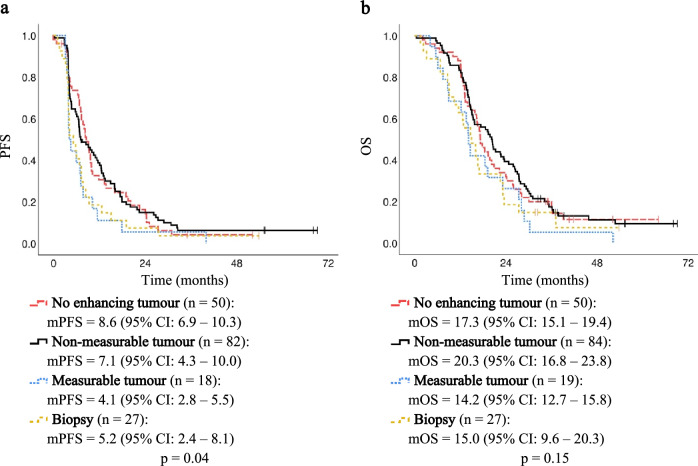
Kaplan–Meier plots with median progression-free survival (mPFS) (**a**) and median overall survival (mOS) (**b**) for patients with either no contrast-enhancing residual tumour, non-measurable contrast-enhancing residual tumour, measurable contrast-enhancing residual tumour, or biopsy. The vertical markers indicate censored patients. Median PFS and OS with 95% confidence intervals (CI) are presented below each plot.

In summary, the only candidate factor consistently associated with both progression-free survival and overall survival was distant tumours. In the Kaplan–Meier analyses, patients with distant tumours had significantly worse progression-free and overall survival over time than patients without distant tumours (Fig. 2a and b). Differences in survival over time with resection status on postoperative MRI are presented in Fig. 3a and b and were only significant for progression-free survival. The survival over time was similar between no- and non-measurable contrast-enhancing residual tumours and between measurable tumours and biopsy, and when these were compared as two groups, both the progression-free and overall survival over time significantly improved (Supplementary Tables S3 and S4).

### Multivariate analyses

Among the candidate factors screened by univariate analysis, distant tumour and resection status were associated with survival (p < 0.10) and were considered for multivariate analysis. In our analysis, distant tumour and multifocal disease were closely correlated (rank correlation of 0.52). When both factors were included in the multivariate model, multifocal disease was not associated with progression-free survival (p = 0.32) or overall survival (p = 0.18), while distant tumours remained significantly associated with overall survival. Therefore, distant tumours, in preference to multifocal disease, were selected for the final multivariate analysis, as shown in Table 2.

**Table 2:** Multivariate analysis.

		PFS	OS
Age, per 10-year increase		1.05 [0.89–1.25] p = 0.55	0.88 [0.74–1.05] p = 0.15
Methylated MGMT		0.47 [0.33–0.66] **p < 0.001**	0.38 [0.26–0.56] **p < 0.001**
Corticosteroid use		1.09 [0.78–1.52] p = 0.60	1.16 [0.82–1.65] p = 0.40
Distant tumour (ep-MRI)		2.25 [1.32–3.86] **p = 0.003**	2.81 [1.69–4.68] **p < 0.001**
Performance status (0 as reference)	1	1.15 [0.82–1.63] p = 0.41	1.37 [0.95–1.96] p = 0.09
	2	0.94 [0.42–2.09] p = 0.88	1.51 [0.64–3.56] p = 0.35
Resection (biopsy as reference)	No contrast-enhancing tumour	0.59 [0.36–0.96] **p = 0.04**	0.67 [0.40–1.11] p = 0.12
	Non-measurable tumour	0.53 [0.33–0.86] **p = 0.01**	0.53 [0.32–0.87] **p = 0.01**
	Measurable tumour	0.96 [0.49–1.89] p = 0.91	0.86 [0.44–1.67] p = 0.65

Significant values are in bold.

In the multivariate analysis, distant tumours were significantly associated with poor progression-free survival (HR = 2.25, 95% CI 1.32–3.86, p = 0.003). Factors associated with improved progression-free survival were methylated MGMT (HR = 0.47, 95% CI 0.33–0.66, p < 0.001) as well as having no contrast-enhancing residual tumour (compared with biopsy) (HR = 0.59, 95% CI 0.36–0.96, p = 0.04) or non-measurable contrast-enhancing residual tumour (compared with biopsy) (HR = 0.53, 95% CI 0.33–0.86, p = 0.01). Increased age, corticosteroid use, performance status and measurable contrast-enhancing residual tumour (compared with biopsy) did not significantly influence progression-free survival. The concordance index (C-index) for the multivariate analysis of progression-free survival was 0.63.

Factors associated with poor overall survival were the presence of distant tumours on postoperative MRI (HR = 2.81, 95% CI 1.69–4.68, p < 0.001). Improved overall survival was seen with methylated MGMT (HR = 0.38, 95% CI 0.26–0.56, p ≤ 0.001) and non-measurable contrast-enhancing residual tumour (compared with biopsy) (HR = 0.53, 95% CI 0.32–0.87, p = 0.01). Increased age, corticosteroid use, performance status, no contrast-enhancing residual tumour and measurable contrast-enhancing residual tumour (compared with biopsy) did not significantly influence overall survival. The C-index for the multivariate analysis of overall survival was 0.66.

Tenfold cross validation confirmed the results of the final model for progression-free and overall survival shown in Table 2, with a C-index corrected for optimism of 0.65 and 0.61 respectively.

## Discussion

We examined the prognostic value of the radiological classification of residual tumour and contrast enhancements on early postoperative MRI following glioblastoma surgery. The presence of tumours distant to the resection cavity were significantly associated with poor progression-free and overall survival, while patients with non-measurable contrast-enhancing residual tumour on postoperative MRI were associated with improved survival. However, the morphology of contrast enhancements was not significantly associated with survival. The results underline the importance of selecting appropriate radiological imaging features for modelling of the prognosis for patients with glioblastoma.

The relation between the presence of tumours distant from the resection cavity on postoperative MRI and patient survival has not been previously investigated. Distant tumours on the postoperative MRI either represented novel tumour components not seen on the preoperative MRI (7 of 22 patients) or tumour components which had been visible on the preoperative MRI but not removed during surgery (15 of 22 patients). As a result, a significant correlation between the presence of distant tumours and multifocal disease is expected. However, it is noteworthy that in the multivariate analysis involving both factors, only distant tumours maintained a significant association with survival. This implies that the detection of distant tumours on postoperative MRI may have a stronger prognostic impact than multifocal disease identified on preoperative MRI.

The results of our study consistently demonstrate a negative impact on both progression-free and overall survival of distant tumours with major implications for patient prognosis (e.g. shortening of OS from 20.3 to 13.5 months in Kaplan–Meier analysis). These findings were significant in the multivariate analyses despite the low number of patients (22 of 153). Our results suggest that the presence of distant tumour on postoperative MRI is an important factor that should be considered in future prognostic models.

Previous studies have demonstrated that some postoperative contrast enhancements are surgically induced, appearing with increasing frequency and intensity in the days and weeks following surgery, and are sometimes difficult to distinguish from residual tumours^17,22,28^. Thin linear contrast enhancements have been previously associated with surgically induced enhancements^18,21^, and in a study by Majos et al.^23^, thin linear enhancements were found to be favourable to thick-linear or nodular enhancements in relation to survival. However, our study did not find any association between different patterns of contrast enhancement (no enhancement, thin linear, diffuse, thick linear, nodular) and survival in the univariate analyses (Supplementary Table S1).

The relationship between the extent of resection and the survival of glioblastoma patients has been the subject of extensive research. Previous studies mostly agree that a greater extent of resection is favourable for survival^13,29–31^. However, to our knowledge, the prognostic value of resection status as evaluated by the RANO-working group classification has not been examined, despite being used as a baseline for the assessment of tumour progression^15^. Significantly improved progression-free survival was seen for patients with no contrast-enhancing residual tumour and non-measurable contrast-enhancing residual tumour when compared with patients receiving only a biopsy, while for overall survival, only patients with non-measurable contrast-enhancing tumour fared better than patients receiving only a biopsy. Unexpectedly, there was no significant association (HR = 0.67, p = 0.12) between the absence of contrast-enhancing residual tumour and overall survival. These findings differ somewhat from the results of other studies, which state that total or near total resection leads to longer survival compared to biopsy, as summarised by Brown et al.^13^. Considering this, the limited sample size may play a role in our results. Also, it is possible that the RANO-working group classification is too crude and that a volumetric definition of residual tumour may be more appropriate, as indicated by recent studies^32–34^.

The final multivariate prognostic model confirmed methylated MGMT as being an independent prognostic factor associated with improved survival. In contrast to previous prognostic models for patients with glioblastoma^5–7^, age was not associated with survival. This observation may be explained by improved selection of patients benefitting from standard therapy during the study period, whereby elderly patients or patients with poor performance status were more frequently prescribed palliative regimens (e.g. short-course radiotherapy with or without temozolomide depending on MGMT status)^4^. In addition, the exclusion of IDH mutated astrocytoma grade 4 patients, who are typically younger and have a better prognosis may have contributed to the lack of association between age and survival. Furthermore, corticosteroid use and performance status were not significantly associated with survival which may be explained by the small sample of the study. Consistent with prior studies^6,7^, our prognostic model demonstrated a C-index of 0.63–0.66 indicating a 63–66% probability of agreement between predicted and observed survival. This implies that the model can predict survival with a reasonable precision but unknown prognostic factors may remain.

This study suffers from several limitations that should be considered when interpreting the results. First, the study is limited by the single centre study design and the sample size. Second, this is an observational study and therefore is susceptible to biases that cannot be controlled for, such as confounding and selection bias. However, efforts were made to minimise these biases by using a consecutive cohort of patients with glioblastoma treated with standard therapy and adjusting for known independent prognostic factors. Additionally, the overall survival for our study cohort was comparable to our published consecutive database matched by year of inclusion (HR = 1.02, p = 0.86)^5^, suggesting that our study was not influenced by selection bias. Nevertheless, selection bias in addition to the smaller sample size and the added covariates, may explain the lack of significant association for some of our previously reported independent prognostic factors^5^. Finally, in the definition of distant tumours, only the distance (1 cm) from the resection cavity was considered. However, due to the limited number of patients with distant tumours on postoperative MRI, separate contrast-enhancing areas connected by non-enhancing tumour on T2/FLAIR were still considered distant tumours and is a limitation of this study.

The findings of this study are particularly significant in the context of ongoing efforts to develop a surgical classification system for glioblastoma based on postoperative MRI^32^. The negative impact of distant tumours on patient survival suggests that any such classification system should consider not only the resection cavity, but also any other residual tumour foci visible on the early postoperative MRI. The clinical usefulness of these findings should be further investigated to improve patient outcomes.

In this study of 187 glioblastoma patients treated with standard oncological therapy following glioblastoma surgery, we found that the presence of distant tumours on postoperative MRI was significantly associated with poor survival. Surgical resection resulting in a non-measurable contrast-enhancing residual tumour on postoperative MRI was associated with improved survival. However, the morphology of contrast enhancements (thin linear, thick linear, diffuse, nodular) was not associated with survival.

### Supplementary Information

Supplementary Tables.

## Data Availability

The data presented in this study are available on reasonable request from the corresponding author.
