# Supplementary material for: Prognostic relevance of radiological findings on early postoperative MRI for 187 consecutive glioblastoma patients receiving standard therapy
Source: Sci Rep. 2024 May 14;14:10985. doi: 10.1038/s41598-024-61925-3 (PMC11094076; doi:10.1038/s41598-024-61925-3)
Supplement: Supplementary file 1 — Supplementary Tables. [file 41598_2024_61925_MOESM1_ESM.pdf]

Supplementary Document

Prognostic Relevance of Radiological Findings on Early Postoperative MRI for 187 Consecutive Glioblastoma Patients Receiving Standard Therapy

Alexander Malcolm Rykkje[1,5,*], Jonathan Frederik Carlsen[1,5], Vibeke Andrée Larsen[1], Jane Skjøth-Rasmussen[2,4,5], Ib Jarle Christensen[4], Michael Bachmann Nielsen[1,5], Hans Skovgaard Poulsen[3,4], Thomas Haargaard Urup[3,4,+], and Adam Espe Hansen[1,4,5,+]

[1]Department of Radiology, Rigshospitalet, Copenhagen, Denmark. [2]Department of Neurosurgery, Rigshospitalet, Copenhagen, Denmark. [3]Department of Oncology, Rigshospitalet, Copenhagen, Denmark. [4]The DCCC Brain Tumor Center, Rigshospitalet, Copenhagen, Denmark. [5]Department of Clinical Medicine, University of Copenhagen, Copenhagen, Denmark.

[*]e-mail: alexander.rykkje@regionh.dk

[+]these authors contributed equally to this work

Supplementary Table S1. Univariate COX-analyses for contrast enhancements

Covariate	Progression-free survival (HR) [95% CI]	Overall Survival (HR) [95% CI]
**Contrast enhancements (excluding measurable and distant tumours; CE stratified; MRI with no contrast enhancement as reference)**		
Thin Linear CE	1.40 [0.78 – 2.51]	1.12 [0.61 – 2.06]
	p = 0.27	p = 0.73
Diffuse CE	0.78 [0.33 – 1.87]	0.79 [0.32 – 1.90]
	p = 0.58	p = 0.59
Thick Linear CE	1.11 [0.60 – 2.07]	0.68 [0.36 – 1.32]
	p = 0.73	p = 0.26
Nodular CE	1.23 [0.69 – 2.20]	1.07 [0.59 – 1.93]
	p = 0.48	p = 0.83

A total of 118 patients qualified for analysis (of the 153 patients receiving postoperative MRI, 35 patients with measurable or distant tumours were excluded). Results were stratified in order of perceived severity (From less severe: no contrast enhancement -> linear enhancement -> diffuse enhancement -> thick linear enhancement -> nodular enhancement). MRI with no contrast enhancement was used as reference.
Abbreviations: CE, contrast enhancement.

Supplementary Table S2. Univariate COX-analyses for other variables

**Covariate**	**Progression-free survival (HR) [95% CI]**	**Overall Survival (HR) [95% CI]**
**Other variables**		
Age, per 10-year increase	0.91 [0.78 – 1.06]	0.80 [0.68 – 0.94]
	p = 0.21	**p = 0.01**
Gender (female *vs.* male)	0.92 [0.68 – 1.24]	1.01 [0.75 – 1.38]
	p = 0.58	p = 0.93
Corticosteroid use (yes *vs.* no)	1.19 [0.88 – 1.62]	0.96 [0.71 – 1.31]
	p = 0.25	p = 0.82
Multifocal *vs.* single lesion	1.77 [1.15 – 2.72]	2.28 [1.49 – 3.47]
	**p = 0.01**	**p < 0.001**
ECOG performance status (0 as reference)		
ECOG performance status affected (PS = 1)	1.01 [0.73 – 1.39]	1.11 [0.80 – 1.53]
	p = 0.96	p = 0.54
ECOG performance status affected (PS = 2)	0.82 [0.38 – 1.80]	1.01 [0.44 – 2.31]
	p = 0.60	p = 0.98
MGMT methylated *vs.* *un*methylated	0.50 [0.37 - 0.69]	0.46 [0.33 – 0.63]
	**p < 0.001**	**p < 0.001**
Abbreviations: MGMT, O6-methylguanine-DNA methyltransferase; ECOG, eastern corporative oncology group.		

Supplementary Table S3. Univariate COX-analyses for resection status and biopsy

Covariate	Progression-free survival (HR) [95% CI]	Overall Survival (HR) [95% CI]
**Resection status and biopsy**		
No enhancing tumour (biopsy as reference)	0.64 [0.40 – 1.04]	0.74 [0.45 – 1.23]
	**p = 0.07**	p = 0.25
Non-measurable tumour (biopsy as reference)	0.63 [0.40 – 0.98]	0.68 [0.43 – 1.09]
	**p = 0.04**	p = 0.11
Measurable tumour (biopsy as reference)	1.10 [0.60 – 2.01]	1.11 [0.61 – 2.03]
	p = 0.75	p = 0.73
Overall p-value	**p = 0.06**	p = 0.18
**Resection status and biopsy (grouped)**		
No contrast-enhancing tumour or non-measurable tumour vs measurable tumour or biopsy	0.61 [0.43 – 0.86]	0.67 [0.47 – 0.96]
	**p = 0.01**	**p = 0.03**

Supplementary Table S4. Kaplan-Meier analyses: Progression-free survival and Overall survival for resection status and biopsy, grouped

	N (PFS / OS)	Progression-free survival (median) [95% CI]	Overall Survival (median) [95% CI]
**Resection status and biopsy (grouped)**			
No contrast-enhancing tumour or non-measurable tumour vs measurable tumour or biopsy	132 / 134	8.35 [6.52 – 10.17]	19.25 [16.64 – 21.86]
	45 / 46	4.57 [2.70 – 6.44]	14.69 [11.96 – 17.42]
		**p = 0.004**	**p = 0.03**
